# The Medical Association

**Published:** 1894-04

**Authors:** 


### *EDITORIAL.*

The office of THE JOURNAL is in room 330 Equitable Building.

Address all communications, and make all remittances payable to THE ATLANTA MEDICAL AND SURGICAL JOURNAL, Atlanta, Ga.

Articles for publication should reach this office not later than the 15th instant.

The editors are not responsible for views expressed by contributors.

### THE MEDICAL ASSOCIATION.

The State Medical Association will meet in Atlanta on the 18th 19th and 20th of the month. It is the wish of the officers of the Association and of the profession in Atlanta that all members who can do so will be present at this meeting. Elsewhere will be found a partial list of the papers to be read. Several prominent gentlemen from a distance have promised to attend and present papers.

We are assured that this meeting will be unusually interesting and well attended. At this juncture in the history of the Association, when an effort is being made to retrieve some of its lost prestige it is important that the representative men of the State attend its meetings and participate in its discussions. It is every one's medical duty to assist and encourage the work of rehabilitation.

Let all come to Atlanta who can. You will be well taken care of, and your stay in the Gate City will be both pleasant and profitable.

